# Intracerebral pressure waves and sleep disordered breathing

**DOI:** 10.1186/2045-8118-12-S1-O67

**Published:** 2015-09-18

**Authors:** Aruna S Rao, David Solomon, Abhay R Moghekar

**Affiliations:** 1 The Johns Hopkins Hospital, USA

## Introduction

Increases in Intracerebral pressure (ICP) have been reported to occur in patients with sleep disordered breathing (SDB) in association with the periods of oxygen desaturation. These studies however, involved very small sample sizes.

## Objective

The purpose of our study was to assess the relationship between alterations in ICP and oxygen desaturations using a large cohort of patients undergoing continuous Spinal fluid pressure monitoring for evaluation of hydrocephalus.

## Methods

Patients admitted to the CSF disorders unit for continuous spinal fluid pressure monitoring were selected for analysis. The pressure waves were recorded via pressure transducer attached to an indwelling Codman spinal catheter. Oxygen saturation was recorded by pulse oximetry. The Spinal fluid pressure waves and oxygen saturation waveforms were converted to digital format for analysis. Our outcome variables were 1. Oxygen desaturation defined as 4% below or 96% of the mean 2. Elevated ICP defined as values above the median value for each patient.

We dichotomized the Oxygen data into normal or desaturations and ICP data to above or below the median ICP. We computed the association between Dichotomized Oxygen saturation versus dichotomized ICP using the chi square test. We analyzed desaturations with ICP as a continuous variable using the t test.

## Results

A consecutive series of 100 patients (median age was 61.5 years; 58% were women) were analyzed. We found that oxygen saturation was more likely to be low immediately after an instance of elevated ICP and ICP is significantly higher immediately before periods of oxygen desaturation. This finding was statistically significant association (p < 0.05) in 39 (39%) of patients. A diagnosis of NPH did not differ between the 2 groups (p=0.2) but a confirmed diagnosis of SDB did (p=0.07).

## Conclusion

In patients with continuous spinal fluid pressure monitoring, small but consistent elevations in spinal fluid pressure are associated with oxygen desaturations in patients with sleep disordered breathing.

